# ERα36-High Cancer-Associated Fibroblasts as an Unfavorable Factor in Triple-Negative Breast Cancer

**DOI:** 10.3390/cancers14082005

**Published:** 2022-04-15

**Authors:** Anna Nagel, Marta Popeda, Anna Muchlinska, Rafal Sadej, Jolanta Szade, Jacek Zielinski, Jaroslaw Skokowski, Magdalena Niemira, Adam Kretowski, Aleksandra Markiewicz, Anna J. Zaczek

**Affiliations:** 1 Laboratory of Translational Oncology, Intercollegiate Faculty of Biotechnology, Medical University of Gdansk, 80-211 Gdansk, Poland; anna.nagel@gumed.edu.pl (A.N.); marta.popeda@gumed.edu.pl (M.P.); anna.jurek@gumed.edu.pl (A.M.); aleksandra.markiewicz@gumed.edu.pl (A.M.); 2 Laboratory of Molecular Enzymology and Oncology, Intercollegiate Faculty of Biotechnology, Medical University of Gdansk, 80-211 Gdansk, Poland; rafal.sadej@gumed.edu.pl; 3 Department of Pathomorphology, Medical University of Gdansk, 80-211 Gdansk, Poland; jszade@gumed.edu.pl; 4 Department of Surgical Oncology, Medical University of Gdansk, 80-211 Gdansk, Poland; jacek.zielinski@gumed.edu.pl (J.Z.); jskokowski@gumed.edu.pl (J.S.); 5 Department of Medical Laboratory Diagnostics-Biobank Fahrenheit BBMRI.pl, Medical University of Gdansk, Debinki Street 7, 80-211 Gdansk, Poland; 6 Clinical Research Centre, Medical University of Bialystok, 15–276 Bialystok, Poland; magdalena.niemira@umb.edu.pl (M.N.); adamkretowski@wp.pl (A.K.)

**Keywords:** cancer-associated fibroblasts, breast cancer, estrogen receptor alpha 36, triple-negative breast cancer

## Abstract

**Simple Summary:**

The tumor microenvironment, as an important constituent of neoplastic tissue, has been a promising target for cancer therapy. Triple-negative breast cancer accounts for around 10–20% of invasive breast cancers. This study describes a new cancer-associated fibroblast subtype characterized by high ERα36 levels that secretes HGF, which can impact triple-negative breast cancer. The data enlighten the importance of the stromal effect on the disease course and underlines the significance of further research on the tumor microenvironment and its role in the progression of cancer.

**Abstract:**

Background: Cancer-associated fibroblasts (CAFs) are the most abundant cell type in the tumor microenvironment (TME). Estrogen receptor alpha 36 (ERα36), the alternatively spliced variant of ERα, is described as an unfavorable factor when expressed in cancer cells. ERα can be expressed also in CAFs; however, the role of ERα36 in CAFs is unknown. Methods: Four CAF cultures were isolated from chemotherapy-naïve BC patients and characterized for ERα36 expression and the NanoString gene expression panel using isolated RNA. Conditioned media from CAF cultures were used to assess the influence of CAFs on triple-negative breast cancer (TNBC) cells using a matrigel 3D culture assay. Results: We found that ERα36^high^ CAFs significantly induced the branching of TNBC cells in vitro (*p* < 0.001). They also produced a set of pro-tumorigenic cytokines compared to ERα36^low^ CAFs, among which hepatocyte growth factor (HGF) was the main inducer of TNBC cell invasive phenotype in vitro (*p* < 0.001). Tumor stroma rich in ERα36^high^ CAFs was correlated with high Ki67 expression (*p* = 0.041) and tumor-associated macrophages markers (CD68 and CD163, *p* = 0.041 for both). HGF was found to be an unfavorable prognostic factor in TCGA database analysis (*p* = 0.03 for DFS and *p* = 0.04 for OS). Conclusions: Breast cancer-associated fibroblasts represent distinct subtypes based on ERα36 expression. We propose that ERα36^high^ CAFs could account for an unfavorable prognosis for TNBC patients.

## 1. Introduction

Triple-negative breast cancer (TNBC) accounts for around 10–20% of invasive breast cancers [1]. Its aggressive behavior and lack of specific treatment contribute to the highest mortality among all breast cancer subtypes, which is estimated at around 20% [1,2,3]. TNBC is characterized by the lack of estrogen receptor (ER), progesterone receptor (PR), and by the lack of human epidermal growth factor (HER2) amplification/overexpression, therefore reducing the options of targeted therapies [4].

Recent findings propose cancer-associated fibroblasts (CAFs) as a potential therapy target for breast cancer (BC) [5,6,7]. CAFs are spindled-shaped cells, and as the most abundant cell type in the tumor microenvironment, they interact closely with cancer cells and contribute to tumorigenesis [8]. Activated fibroblasts within the tumor exhibit enhanced proliferation, migration, and elevated secretion levels of chemokines and growth factors [9,10]. The emerging evidence suggests that CAF populations are highly heterogeneous because of their various origins, including residual quiescent fibroblasts, pericytes, or bone marrow-derived progenitor cells [6,10]. CAF subpopulations are known to exert different, sometimes contradictory roles in cancer [8,11]. For example, CD146^pos^ CAFs in ER-positive BC maintain ER expression in cancer cells and sensitivity to tamoxifen treatment, whereas CD146^neg^ CAFs suppress the expression of ER and promote tamoxifen resistance [12]. In TNBC, stroma-rich tumors in hematoxylin and eosin staining were correlated with a shorter relapse-free period and poor overall survival. They were also an independent prognostic factor for the total group of patients in the study [13].

Interestingly, ER itself can be expressed by CAFs, what has functional consequences. In prostate cancer (PCa), ER-positive CAFs correlated with better patient outcomes and lower invasiveness, which is linked to the downregulation of MMP3 [14]. ER-positive CAFs also suppress PCa cell invasion by the reduction of CCL5 and IL-6 secretion, as well as macrophage infiltration [15]. A study by Da et al. indicated the pro-proliferative action of ER-positive CAFs in PCa in vitro and in a mouse model [16]. In cervical cancer, an estrogen receptor was also described as a modulator of CAF function. ER-antagonists (fulvestrant and methyl piperidino pyrazole) downregulated the expression of genes associated with cell cycle and metabolism in CAFs, which affected tumor progression [17]. In breast cancer, however, there are no data about the ER role in CAFs. Hence, exploring this marker also in tumor stroma may open further opportunities for cancer treatment.

ERα36 is an alternative estrogen receptor alpha isoform coded by the *ESR1* gene [18]. It lacks transcriptional activation domains but retains the DNA-binding domain [19,20]. In breast cancer, ERα36 is described as a rapid activator of non-genomic ER signalization via the MAPK signaling pathway, which leads to uncontrolled proliferation and anti-apoptotic events [21]. Our previous study showed that a high expression of ERα36 is an unfavorable prognostic factor in breast cancers, also in the ER-negative group [22]. At the same time, 22% of stromal cells in ER-negative cancers were positive for ERα36. Therefore, we decided to investigate the role of ERα36 in the most abundant stromal cells in breast cancer—cancer-associated fibroblasts—to check if they might play a role in engaging malignant phenotype in one of the most aggressive breast cancer subtypes—triple-negative cancer.

## 2. Materials and Methods

### 2.1. Cell Lines, Antibodies, and Reagents

MDA-MB-231 and Hs 578T cells were purchased from the American Tissue Culture Collection (ATCC, Manassas, VA, USA). The cells were passaged for a maximum of 3 to 4 months post-resuscitation and routinely tested for mycoplasma contamination. The cells were cultured in DMEM supplemented with 10% fetal bovine serum (FBS). All the media and their supplements were purchased from GE Healthcare HyClone or Sigma Aldrich (Saint Louis, MO, USA). All the cells were cultured up to 90–95% confluency and the media were changed every 2–3 days. The following antibodies were purchased: from Cell Signaling Technology—anti-c-Met-Tyr1234/1235 (#3077), anti-c-Met (#4560), anti-Akt-Ser473 (#4058), anti-Akt (#9272), anti-ERK1/2-Thr202/Tyr204 (#9101), anti-ERK (#4695); from Dako Agilent—anti αSMA (IR611), from Novus Biologicals—anti-vimentin (NBP1–31327), from Cell Applications—anti-ERα36 (CY-1109), from BD—anti-E-cadherin (Clone 36, 610181), from Abcam—secondary anti-rabbit IgG DyLight 488 (ab96883), secondary anti-mouse IgG DyLight 594 (ab96873), from Sigma Aldrich—β-actin (AC-15) secondary anti-rabbit HRP-conjugated (A9169), secondary anti-mouse HRP-conjugated (A9044). Cytokines were purchased from STI–VEGF (CYT-10–10), IL-8 (chm-231-a), HGF (cyt-090-a), GM-CSM (cyt-221-a), CXCL1 (100–031S), and Peprotech-CXCL5 (300–22). HGFR inhibitor-capmatinib were purchased from Selleckchem (Cat. No. S2788).

### 2.2. Cancer-Associated Fibroblast Isolation and Conditioned Media Preparation

Tumor tissues were collected from patients treated at the Medical University of Gdansk. The study was approved by the Ethical Committee of the Medical University of Gdansk (NKBBN 94/2017). Informed consent was obtained from all subjects involved in the study. The tissue was collected into cold DMEM medium supplemented with 10% FBS and an antimycotic/antibiotic mix (Sigma Aldrich, A5955) and stored at 2–8 °C until ready for processing, but for no longer than 4 h. After mincing, the tissue was digested in 0.35 mg/mL collagenase (Sigma Aldrich, C2674) and 0.35 mg/mL hyaluronidase (Sigma Aldrich, H3506) solution in PBS for 1 h with rotation at 37 °C and 5% CO2. Then, the suspension was centrifuged 400× *g* for 5 min, the pellet was suspended in DMEM medium supplemented with 10% FBS and antimycotic/antibiotic mix and transferred to the cell culture dish. The CAFs were serially trypsinized to continually reduce the tumor cell populations. The serial trypsinization method utilizes the differences in the detachment times of various cell types, CAFs detach from the tissue culture-treated plastic faster than cancer cells, and after 2–3 passages, CAF culture should be homogeneous [23]. The homogeneity was checked by immunofluorescence staining using CAF markers—vimentin (VIM), alpha smooth muscle actin (αSMA), and tumor cells marker—E-cadherin as a control. All CAF experiments were conducted using cells between the 3rd and 10th passages. For the conditioned media collections, the media was changed in the cell cultures with 70–80% confluency and collected after 72 h. Media from different pass times were collected, frozen at −80 °C, and mixed before use to assure consistency.

### 2.3. Immunofluorescence Staining

Cells were seeded on the sterilized cover glass and, after 24 h, were fixed and permeabilized using a methanol/acetone mix for 15 min. For blocking, 5% BSA in PBS was used. Primary antibodies were diluted in Antibody Diluent (Dako Agilent, Santa Clara, CA, USA) and incubated with cells for 30 min. Imaging was performed using an Olympus IX83 fluorescent microscope and CellSens Imaging Software (Olympus Life Science, Waltham, MA, USA).

### 2.4. Western Blotting

Cell lysates were prepared using RIPA buffer with protease and phosphatase inhibitors (Sigma Aldrich, Saint Louis, MO, USA, and Thermo Fisher Scientific, Waltham, MA, USA). SDS-PAGE was performed using TGX gels for 1 h and 250 V (Bio-Rad, Hercules, CA, USA), then the proteins were transferred onto the PVDF membrane by semi-dry transfer (Bio-Rad, Hercules, CA, USA). The membranes were blocked with 5% skimmed milk and probed with specific antibodies overnight at 4 °C. Secondary antibodies conjugated with HRP (Sigma-Aldrich) and Western Lightning Plus-ECL (Amersham) were used to visualize specific protein bands. Original images about western blotting can be found at Supplementary Materials, File S1. 

### 2.5. Cancer-Associated Fibroblasts Secretome Analysis

Cytokine detection in the CAF-conditioned media was performed using a Proteome Profiler Human XL Cytokine Array Kit (R&D Systems) according to the manufacturer’s protocol. For this experiment, phenol red-free DMEM medium supplemented with 10% FBS was used.

### 2.6. Cell Migration Assay

Two hundred thousand MDA-MB_231 cells were seeded into a Boyden chamber with 8µm pores and inserted into the well with the CAF-conditioned media. The cells were incubated for 16 h. Then cells from the inner side of the chamber were scrubbed off, and the cells on the other side were stained with Hoechst. Migrated cells were observed using a fluorescent microscope.

### 2.7. Analysis of Cell Growth in Three-Dimensional Cultures

The cells (1.5 × 10^3^ MDA-MB-231 or 2 × 10^3^ Hs 578T) were resuspended in 40 µL of growth factor-reduced matrigel mixed 1:1 with medium and incubated in a humidified incubator at 37 °C for up to 60 min to solidify. Two drops of cell suspension were plated on a tissue culture plate for each sample. Solidified matrigel drops were covered with the appropriate growth media with or without supplementation. The medium was replaced every third day. Pictures were taken between days 7 and 14. For all 3D culture experiments, representative pictures were taken using an Olympus IX83 microscope. To evaluate the number of cell colonies branching, three independent visual fields were investigated for each drop, six for each sample. Conditioned media from the CAF cultures were used to analyze the effect of CAFs on MDA-MB-231 invasiveness. Media supplemented with appropriate cytokines were used to detect the one that is responsible for that phenotype. All 3D experiments were performed in triplicate, and the means from all the experiments are shown in graphs.

### 2.8. Stimulation with CAF-Conditioned Media and HGF, Signaling Analysis, and Inhibitory Effects

For the analysis of signaling triggered by ERα36^high^ CAFs, cells were routinely starved overnight in serum-free media. Where required, the media were supplemented with a c-Met inhibitor—capmatinib (50 nM)—for 2 h prior to stimulation. The cells were then stimulated with CAFs4-conditioned medium or HGF (10 ng/mL) for indicated time periods. All stimulations were performed in triplicate.

### 2.9. Clinical Data Analysis

A total of 103 primary tumors from breast cancer patients (stages I–III) treated at the Medical University of Gdansk were investigated. Their detailed clinical characteristics are listed in Table 1. The study was granted permission from the Bioethical Committee of the Medical University of Gdansk.

### 2.10. ERα36 Protein Levels in Tumor Stroma

Tissue microarrays (TMA) were prepared by sampling up to five non-adjacent tissue cores 1 mm in diameter from each formalin-fixed, paraffin-embedded (FFPE) primary tumor. Serial sections were analyzed by manual immunohistochemical staining with commercially available rabbit antibodies against ERα36 specific to the unique C-terminal sequence (Cell Applications Inc., San Diego, CA, USA, Cat# CY-1109; dilution, 1:800; incubation time, 1 h). Secondary anti-rabbit antibodies conjugated with horseradish peroxidase (HRP) were used together with the Novolink Max-Polymer Detection System (Leica Novocastra, Wetzlar, Germany) for the detection of the ERα36 protein. The intensity and the percentage of positively stained fibroblasts were evaluated; since the intensity (score of 1–3) was equal in all samples (2), only the percentage was taken into consideration.

### 2.11. Hormone Receptors and HER2 Status Analysis in Breast Cancer Samples

ER (rabbit monoclonal antibody, clone SP1, Roche, Basel, Switzerland), PgR (rabbit monoclonal antibody, clone 1E2, Roche), and HER2 (rabbit monoclonal antibody, clone 4B5, Roche) were analyzed on the whole slides during the standard pathological assessment of the tumors. TMA, prepared as described above, from PT and LNM were used for SMA (mouse monoclonal antibody, clone 1A4, Dako Agilent, Santa Clara, CA, USA), and antigen retrieval and staining were performed on the automatic devices: BenchMark GX (Roche) for ER, PgR, and HER2 staining, and DAKO AutostainerLink48 (Dako Agilent) for EpCAM and SMA staining. For negative controls, the primary antibodies were omitted. ER, PgR, and HER2 were detected using the UltraView DAB Benchmark XT system (Roche), EpCAM, and SMA with EnVision™ FLEX Dako Autostainer (Dako Agilent). SNAIL staining was performed manually.

### 2.12. Statistical Analysis

The in vitro data were analyzed using Prism 9 software (GraphPad, San Diego, CA, USA). For multiple comparison, Tukey’s test was used. For simple comparison of two normally distributed datasets, Student’s t test was used. Clinical data analysis was performed using Statistica version 12 (StatSoft Dell, Round Rock, TX, USA) software and SPSS Software (IBM, Armonk, NY, USA). Categorical variables were compared by the χ^2^ test. Continuous variables were compared by Spearman’s rank order test. The Mann–Whitney test was used to examine the differences between continuous values in two groups. Kaplan–Meier curves for disease-free survival and overall survival were compared using an F-Cox test.

### 2.13. RNA Extraction

RNA was isolated from the CAFs using RNeasy Mini Kit (Qiagen, Hilden, Germany) according to the manufacturer’s protocol. The RNA concentration and purity were determined using a NanoDrop 1000 spectrophotometer (Thermo Scientific, Wilmington, DE, USA). The RNA extraction from FFPE breast tumor samples was performed as described [24].

### 2.14. nCounter Gene Expression Assay

The CAFs and FFPE RNA samples were analyzed in separate batches. RNA extracted from CAFs (300 ng) was subjected to expression profiling with an nCounter PanCancer Immune Profiling Panel (NanoString Technologies, Seattle, WA, USA) according to the manufacturer’s procedures for hybridization, detection, and scanning. RNA extracted from FFPE tissues was processed as described [24].

### 2.15. NanoString Data Processing

The CAFs and FFPE RNA data were processed in separate batches. For each sample, background correction and normalization were performed using nSolver 4.0 software, as previously described [24]. In brief, the background level was estimated by thresholding over the mean plus 2 standard deviations of the negative control counts. Subsequently, the data were normalized according to the global mean of the counts of positive controls and the most stably expressed housekeeping genes (expression stability assessed with NormFinder)—4 in the FFPE group (SD range of 173.5–228.4 counts) and 18 in the CAF group (SD range of 2.9–66.9 counts). The negative and positive control probes were included in the assay. Following normalization, low-expression genes (log2 mean count in all samples <4 for the CAF dataset and < 6 for the FFPE dataset) were excluded, leaving 326 transcripts in the CAF dataset and 584 transcripts in the FFPE dataset for further analysis. Genes differentiating between ERα36^high^ and ERα36^low^ tumors were selected based on the logarithmic fold change (logFC) calculated for the median normalized counts of each probe in the compared groups. Genes with logFC > 1 were considered upregulated; genes with logFC < −1 were considered downregulated. The differences were estimated with the Mann–Whitney U Test. The data were analyzed using the R statistical environment (3.6.1).

## 3. Results

### 3.1. CAFs Characterized by Different ERα36 Expression Represent Distinct Subpopulations with Diverse Influence on TNBC Cells

Four CAF cell lines were isolated from primary breast tumors (for details, see Section 2). After 3 passages, the cells were checked for CAFs and cancer cell markers—vimentin (VIM), alpha smooth muscle actin (α-SMA), and E-cadherin (E-CAD—by immunofluorescent staining and Western blotting. All obtained CAF cell lines were stained positively for VIM and negatively for E-CAD. In terms of α-SMA, we observed heterogeneity between the obtained cell lines as well as within cells in particular cell lines (Supplementary Materials, Figure S1).

**Figure 1:**
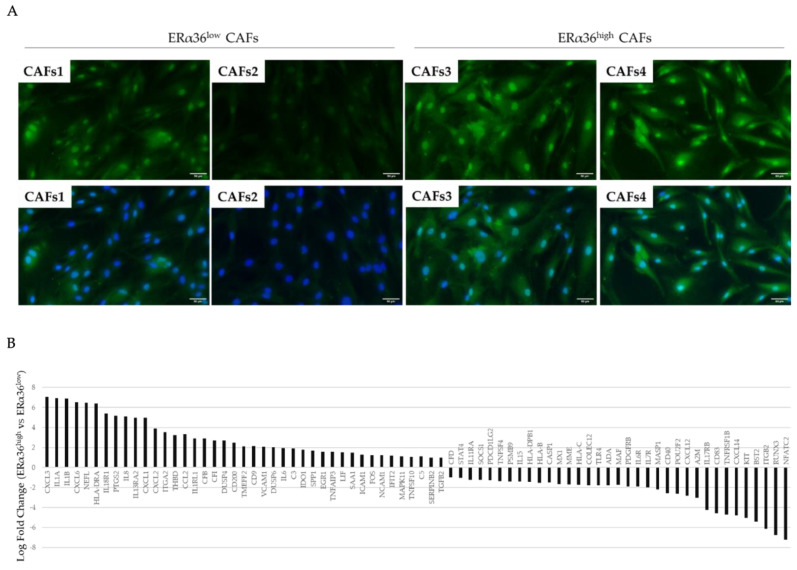

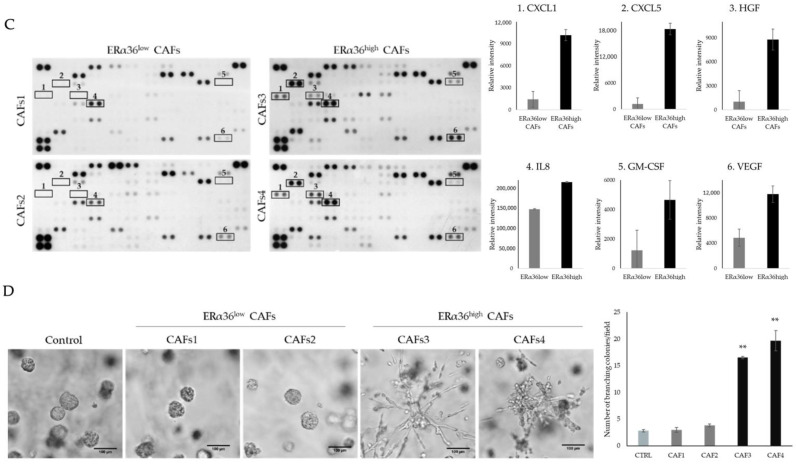
Characterization of CAF cultures from BC patients, (**A**). ERα36 staining (green) by IF and nuclear DAPI staining (blue) of the obtained CAF cell cultures, scale bar: 50 µm. (**B**). nCounter PanCancer Immune Profiling panel analysis of gene expression in ERα36^hihg^ CAFs vs ERα36^low^ CAFs, genes with logFC > 1 (FC—fold change) were considered upregulated, genes with logFC < −1 were considered downregulated, (**C**). Human Cytokine XL Profiler analysis of conditioned media obtained from CAF cultures. The left panel shows secretomes of ERα36^high^ and ERα36^low^ CAFs with marked spots that were secreted differently between the two groups, the right panel quantifies the changes in the six selected cytokines levels, (**D**). 3D matrigel cultures of the TNBC MDA-MB-231 cells treated with the condition media from ERα36^high^ or ERα3 ^low^ CAFs. ERα36^high^ CAF-conditioned media significantly induced branching in MDA-MB-231 cells, ** *p* < 0.0001 calculated vs control; colony photographs were taken using 10× magnification.

The obtained CAF cell lines were stained with a monoclonal anti-ERα36 antibody. Two lines, CAF3 and CAF4, showed significantly higher fluorescence intensity than the CAF1 and CAF2 lines (Figure 1A). Thus, we classified the CAF1 and CAF2 lines as ERα36^low^ and the CAF3 and CAF4 lines as ERα36^high^. Knowing that CAF subpopulations may differ in gene expression as well as in modulation of the immune microenvironment of the tumor [25,26,27,28], we decided to analyze both CAF subpopulations with an nCounter PanCancer Immune Profiling Panel. We found significant differences in gene expression between the ERα36^high^ and ERα36^low^ CAF subpopulations. In total, 41 genes were found to be upregulated (logFC > 1, FC—fold change, the ratio between the ERα36^high^ and ERα36^low^ samples), and 35 were downregulated (logFC < −1) in ERα36^high^ when compared to ERα36^low^ CAFs. The most upregulated genes in ERα36^high^ CAFs were *CXCL3, IL1A, IL1B, CXCL6*, and *NEFL,* while the most downregulated genes were *NFATC2, RUNX3, ITGB2, BST2*, and *KIT* (Figure 1B, Supplementary Data 1).

Differences in the gene expressions of ERα36^high^ and ERα36^low^ CAFs suggest that the subpopulations may also differ in the secretome profiles. Indeed, as revealed by analysis using a Human Cytokine XL Profiler, ERα36^high^ CAFs secreted significantly higher amounts of CXCL1, CXCL5, HGF, IL-8, GM-CSF, and VEGF (Figure 1C). All those cytokines are linked with tumor progression and invasion [28,29,30,31,32,33]; therefore, we tested the influence of CAF secretomes on the 3D growth of triple-negative breast cancer cell line MDA-MB-231. Conditioned media obtained from ERα36^high^ CAFs significantly increased the formation of the branching colonies in growth factor-reduced matrigel (3 and 3.8 branching colonies/visible field for ERα36^low^ CAF1 and CAF2, respectively, vs 16.6 and 19.7 for ERα36^high^ CAF3 and CAF4; Figure 1D). To confirm our findings, we tested CAF-conditioned media on another TNBC cell line—Hs 578T (Supplementary Materials, Figure S2) and observed a similar effect. ERα36-negative CAF-conditioned media induced the branching of the cells in a slower manner than ERα36-positive CAFs (7.5 and 6.2 branching colonies/visible field for ERα36^low^ CAF1 and CAF2, respectively, vs. 15.9 and 15.9 for ERα36^high^ CAF3 and CAF4, respectively). Furthermore, we analyzed the influence of CAF-conditioned media on cell migration. All CAF-conditioned media induced the migration through the Boyden chamber, but with no significant differences between the CAF subtypes (Supplementary Materials, Figure S3).

### 3.2. HFG Secreted by ERα36^high^ CAFs Induces Invasive Phenotype of TNBC Cells

**Figure 2:**
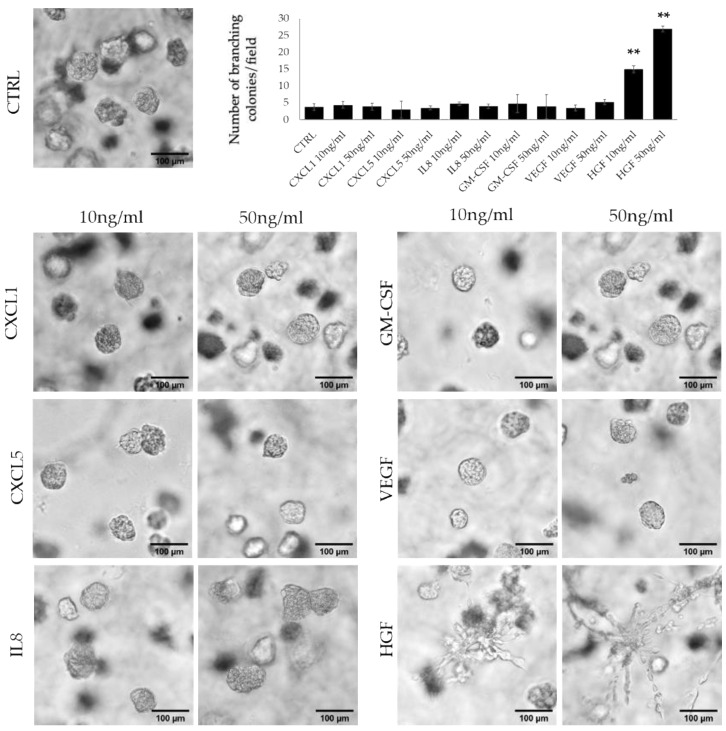
3D matrigel cultures of MBA-MB-231 cells treated with selected cytokines in two concentrations (10 ng/mL and 50 ng/mL), only HGF induced colony branching. ** *p* < 0.001 calculated vs control; culture photographs were taken using 10× objective. The graph represents the number of branching colonies per visible field.

Taking into consideration the differences in the profiles of ERα36^high^ and ERα36^low^ CAF-secreted cytokines, we decided to determine which cytokines are responsible for cell branching in 3D matrigel culture. We tested six cytokines (CXCL1, CXCL5, IL-8, GM-CSF, VEGF, and HGF) in two concentrations, 10 ng/mL and 50 ng/mL, and counted the number of branching colonies per field view. We observed that at both concentrations, only HGF significantly induced colony branching compared to the control (mean of 3.7 colonies/visible field for control vs 14.9 and 26.9 for 10 ng/mL and 50 ng/mL HGF, respectively, *p* < 0.001, Figure 2). HGF at a 50 ng/mL concentration also significantly increased the number of branching colonies compared to 10 ng/mL HGF (14.9 vs 26.9, *p* < 0.001, Figure 2). The results were confirmed on another TNBC cell line—Hs 578T. HGF in both concentrations significantly induced branching of the colonies when compared to the control (8.5 vs. 55.9 for 10 ng/mL, *p* < 0.0001; 8.5 vs. 71.2, *p* < 0.0001). There was also a significant difference between the 10 ng/mL HGF treatment and 50 ng/mL (55.9 vs. 71.2, *p* < 0.0001, Supplementary Materials, Figure S4).

**Figure 3:**
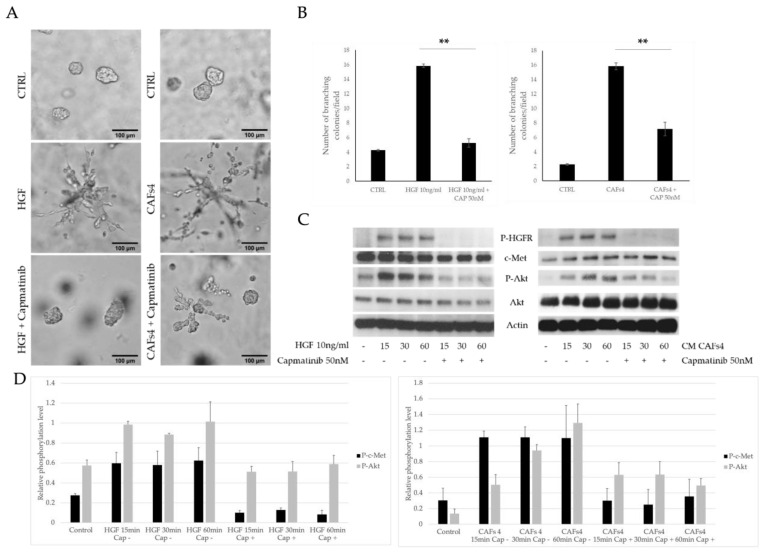
Analysis of c-Met pathway involvement in HGF-mediated MDA-MB-231 colony branching (**A**). Representative colony photographs of 3D matrigel cultures, HGF and HGF with capmatinib treatment on the left panel, conditioned media (CM) from ERα36^high^ (CAF4) culture and capmatinib treatment on the right panel, 10× magnification, capmatinib significantly inhibited colony branching induced by HGF as well as by CAF4-CM (**B**). Graphs representing data from 3D matrigel cultures, ** *p* < 0.005 between HGF or CAF4-conditioned media treatment and addition of capmatinib (**C**). Western blot analysis of c-Met-signaling pathway after HGF and CAF4-CM treatment at different times (15–60 min), with or without capmatinib treatment. Activation of c-Met receptor and Akt was inhibited after capmatinib treatment. (**D**) Densitometry measurements of protein phosphorylation as a ratio between phosphorylated to total protein signal.

Next, we analyzed if the HGF receptor (c-Met, HGFR) is involved in the stimulation of MDA-MB-231 cell branching by conditioned media from CAF cultures. To test this, we used a lower HGF concentration (10 ng/mL, as it still significantly induced colony branching) and c-Met inhibitor-capmatinib [29]. In the 3D matrigel cultures, both HGF and conditioned media from CAF4 culture increased the number of branching colonies (11.75 for HGF and 15.83 for CAF4) in comparison to the control (2.25), and the effect was abolished upon capmatinib treatment of both the HGF group (11.75 for HGF vs. 1.92 colonies for HGF + capmatinib *p* = 0.002) and the CAF4 conditioned media group (15.83 for CAFs vs. 7.17 colonies for CAF4 + capmatinib, *p* = 0.003. Figure 3A,B). Similarly, in the Hs 578T cell line treated with HGF in the presence of capmatinib, a decrease in branching colonies was observed (55.9 vs 6.7, *p* < 0.0001), as well as for the treatment with CAF4-conditioned media with or without 50 nM capmatinib (5.0 vs. 24.1, *p* < 0.001, Supplementary Materials, Figure S5). The above results strongly suggest the involvement of the c-Met signaling pathway in the ERα36^high^ CAF-mediated induction of MDA-MB-231 colony branching. Therefore, we analyzed the phosphorylation of c-Met-related downstream proteins: AKT, FAK, Paxillin, and Scr (Supplementary Materials, Figure S6). We found that both HGF and CAF4-conditioned media induced the phosphorylation of c-Met as well as AKT, which was affected by pretreatment with capmatinib (Figure 3C,D). Taken together, the obtained results suggest that ERα36^high^ CAF subtypes secrete HGF, which induces an invasive phenotype in MDA-MB-231 cells in vitro via the c-Met-AKT pathway.

### 3.3. ERα36^high^ CAFs in Tumor Stroma Correlates with Proliferation and TAMs Markers

**Figure 4:**
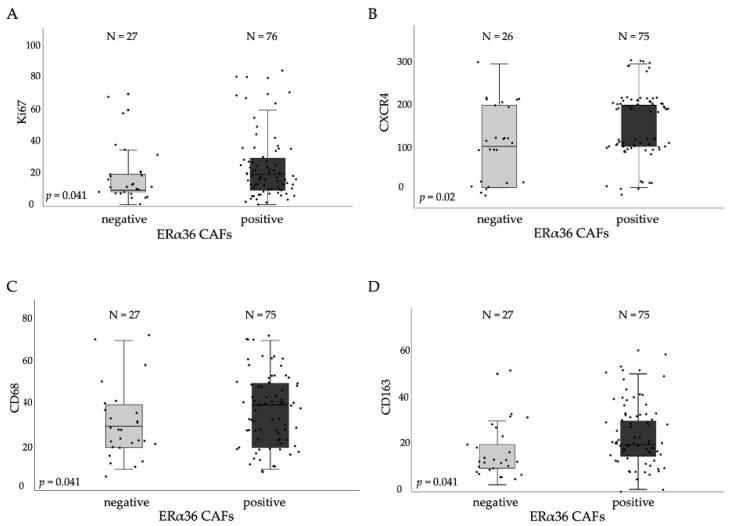
Levels of protein expression in CAFs subgroups, (**A**) Ki67 expression in ERα36 negative and positive CAFs groups, (**B**) CXCR4 expression in ERα36 negative and positive CAFs groups, (**C**) CD68 expression in ERα36 negative and positive CAFs groups, (**D**) CD163 expression in ERα36 negative and positive CAFs groups. Mann-Whitney U test was used in the analysis.

We analyzed by IHC the levels of ERα36 isoform in tumor stromal cells in 103 FFPE breast cancer samples. The mean percentage of positively stained cells was 22.3% (range of 5–62%), and the samples were classified as positive if the percentage of ERα36-positive stroma cells was higher than 12% (cut-off level set at lower quartile value). As a result, 69.1% of all samples were ERα36-positive (representative pictures in Supplementary Materials, Figure S7). We found that ERα36-positive stroma cells correlated positively with Ki67 (median values of 10 vs. 20, *p* = 0.041), CXCR4 (median values of 100 vs. 200, *p* = 0.002), CD68 (median values of 30 vs. 40, *p* = 0.041), and CD163 (median values of 10 vs. 20, *p* = 0.041) protein levels measured by IHC (Figure 4A–D).

### 3.4. Cytokines Produced by ERα36^high^ CAFs Confer Poor Prognosis of TNBC Patients

**Figure 5:**
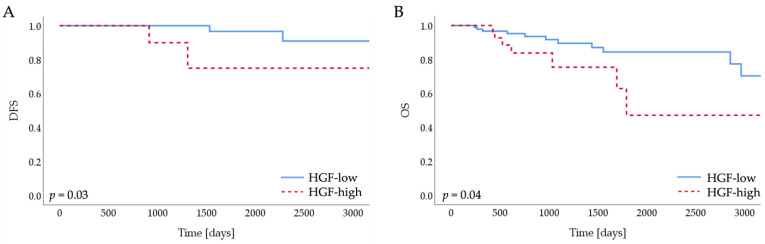
Kaplan–Meier survival curves of (**A**) disease-free survival (DFS) and (**B**) overall survival (OS) according to HGF expression in TCGA database.

Knowing that ERα36^high^ CAFs induce an invasive phenotype of TNBC cells in vitro, ERα36 protein levels in tumor stromal fibroblasts were correlated with clinicopathological data, and protein markers were associated with EMT, stemness, and stroma composition, previously characterized by our team [34,35]. There was no correlation between the ERα36 protein expression and the stage, grade, lymph node status, molecular subtype, or histological type (Table 1) of the analyzed breast tumors. We also found no association between ERα36-positive CAFs and the overall survival of TNBC patients in our group (N = 15, *p* = 0.47, Supplementary Materials, Figure S8). However, the survival analysis might be strongly affected by the very small sample size (N = 14). Taking into consideration that ERα36 isoform expression data are not available in open access databases, we decided to explore the effect of cytokines expressed by ERα36^high^ CAFs on disease-free survival in TNBC using a TCGA database. For all of them, we used the upper quartile cutoff and found that only a high expression of HGF was an unfavorable predictive factor for both disease-free survival and overall survival in TNBC (N = 121, *p* = 0.03 and *p* = 0.04 for DFS and OS, respectively, Table 2, Figure 5A,B).

### 3.5. ERα36 Expression Affects Transcription of Matrix Disassembly and Immune Response Genes in BC Patients

Since tumors with high ERα36 fibroblasts were correlated with higher expressions of CD163 and CD68, which are known to be markers of M2-polarized macrophages, we decided to analyze the expression of immune-related genes in CAFs ERα36^high^ and ERα36^low^ groups of BC patients. We used an nCounter PanCancer Immune Profiling Panel, (NanoString Technologies) in BC patient samples with high and low ERα36 protein levels in tumor stromal fibroblasts. We found that 6 mRNA transcripts were upregulated in the ERα36^high^ group, and 41 transcripts were downregulated. The most upregulated transcript was SPP1 (logFC = 1.64, *p* = 0.01), and the most downregulated mRNA transcript was CXCL13 (logFC = −2.21, *p* = 0.02, Supplementary Data 2).

**Figure 6:**
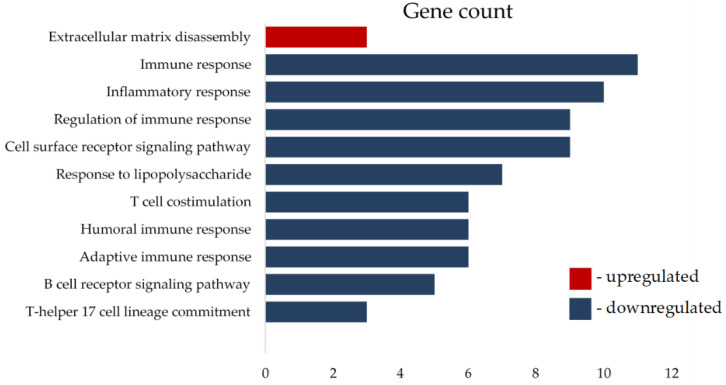
Significantly affected biological processes in BC patients with ERα36high tumor stroma fibroblasts depicted as the number of genes involved in a particular process.

To determine the biological significance of major up- and downregulated transcripts, Gene Ontology Biological Processes were analyzed using the DAVID Functional Annotation Tool. The most upregulated biological process in patients with high levels of ERα36 in tumor stroma fibroblasts was extracellular matrix disassembly (GO:0022617, *q* value = 0.04), and the most downregulated process was the regulation of immune response (GO:0050776, *q* value less than 0.001, Supplementary Data 3). All significantly affected biological processes are listed in Figure 6.

## 4. Discussion

The heterogeneity of CAFs is still a matter of investigation. Here, we presented for the first time that breast cancer-associated fibroblasts express estrogen receptor-α isoform ERα36, and that CAF subpopulations with a high expression of ERα36 secrete a set of cytokines, which are known to have an impact on cancer progression [30,31,32]. Furthermore, in our study, HGF produced by ERα36^high^ CAFs induced an invasive phenotype of TNBC cells in vitro and was correlated with poorer TNBC patient outcomes.

Cytokines produced by ERα36^high^ CAFs—CXCL1, CXCL5, HGF, IL-8, GM-CSF, and VEGF—are mainly pro-tumorigenic. CXCL1 expressed by stromal breast CAFs is correlated with tumor grade, disease recurrence, and decreased patient survival [33]. It is also linked with chemoresistance and metastasis [36]. CXCL5 was identified by Romero-Moreno and colleagues as a key factor in breast cancer cell colonization of the bone in the mouse model [34]. IL-8 was one of the factors to promote TNBC cell colony formation and predict patient survival times [35]. In our analysis, IL-8 was linked with poor overall survival in the TCGA dataset. VEGF is a known pro-angiogenic factor and an unfavorable prognostic marker in breast cancer [37,38]. All the factors identified to be secreted by ERα36^high^ CAFs may affect tumor progression in different ways. However, in our in vitro set up, HGF was a sufficient inducer of MDA-MB-321 cell colonies branching in 3D through the AKT signaling pathway. HGF is known to be broadly expressed by CAFs; however, no study has linked its expression with specific a CAF subtype. In general, HGF-induced c-Met signaling and the associated AKT pathway are known to be tumor-promoting factors in terms of cancer cell proliferation, motility, and invasiveness [30,39,40], which corresponds to our findings. However, one study by Ridolfi and colleagues in 2008 showed the opposite effect of HGF using the same cell line, and it requires further study [41]. The clinical part of our study was mainly limited by the small number of patients with TNBC; thus, we decided to analyze the influence of the ERα36^high^ CAF cytokine panel on BC patients from the TCGA database. HGF was found to confer poor DFS as well as OS in TNBC patients, supporting the results obtained in vitro. These results support other studies where HGF/c-Met signaling has been correlated with poorer patient outcomes and tested as a potential therapy target [42,43]. Our findings suggest ERα36^high^ CAFs as an additional factor in HGF/c-Met signaling activation and tumor progression in TNBC cells.

In the clinical analysis, we found that ERα36-positive fibroblasts correlated positively with CD163 and CD68 expression in BC stroma samples. CD163 and CD68 are factors linked with M2-polarized tumor-associated macrophages (TAMs). Takahashi and colleagues showed that CAFs are able to educate CD14-positive macrophages obtained from healthy donors into pro-tumoral macrophages with high expressions of CD68, CD14, CD163, CD200R, and CD206 [44]. Moreover, the presence of CD163-positive TAMs was recently associated with the poor prognosis of TNBC patients [45]. The above studies have suggested the importance of the CAF–TAM loop in tumor progression. We propose that ERα36^high^ CAFs have their role in macrophages polarization; the understanding of this interaction merits further studies. We also observed high expressions of Ki67 and CXCR4 BC samples in the ERα36^high^ CAF groups, which suggests their role in cancer cell proliferation and migration. Immune-related gene expression and GO biological process analysis revealed that genes overexpressed by the ERα36^high^ CAF group were linked with extracellular matrix disassembly. Indeed, CAFs are known to be the major ECM remodeling agents in TME. However, the most downregulated biological processes were connected with immune and inflammatory responses, as well as with the regulation of immune response. Immune suppression and immune escape are linked with cancer progression and poor patient outcomes [46,47]. Moreover, research data suggest that HGF/c-Met signaling is also involved in the immune response. However, whether it acts as a suppressive or immune-positive stimulus is unknown [48].

During CAF isolation, we found that the obtained cells stained positively for Vimentin and negatively for E-cadherin, in a uniform way. However, alpha-smooth muscle actin (α-SMA) staining was more heterogeneous. This heterogeneity in α-SMA in CAFs has been reported previously, and it has been suggested that CAFs with a high expression of α-SMA represent a pro-tumorigenic population [49,50]. In our study, the heterogeneity of α-SMA staining did not have an impact on any of the obtained results. It also did not correlate with ERα36 expression (data not shown). Due to the heterogeneous α-SMA staining results, we decided to use positive Vimentin staining and negative E-cadherin staining as a CAF marker.

To summarize, we have shown that the heterogeneity of CAFs in breast cancer extends to ERα36 isoform expression. CAFs expressing high levels of ERα36 secrete HGF and induce the aggressive phenotype of TNBC cells in vitro through the activation of the c-Met/Akt pathway, which is known to be involved in cancer progression. High levels of HGF correlated with worse survival of TNBC patients, which might indicate the importance of the stromal effect on the disease course and underlines the importance of further research on the tumor microenvironment and its role in the progression of cancer.

## 5. Conclusions

Our study shows that CAF heterogeneity is a complex phenomenon, and many factors should be taken into consideration during tumor microenvironment investigation. We propose ERα36 as a player in the progression of triple-negative breast cancer.

## Figures and Tables

**Table 1:** Analysis of ERα36 protein levels in tumor stromal fibroblasts in the context of clinicopathological data. Mann–Whitney U test was used in the analysis of two samples, the Kruskal–Wallis test was used in the analysis of three and more samples.

Variable	*n*	Median ERα36 Protein Levels in Tumor Stroma Fibroblasts (25–75th Percentile)	*p*
**T stage**			*p* = 0.151
T1	44	22.25 (15.50–31.00	
T2	53	18.00 (12.25–28.00)	
T3	3	11.25 (9.79–18.13)	
T4	2	7.50 (7.00–8.00)	
**N stage**			*p* = 0.425
N0	43	20.00 (13.33–30.00)	
N1	53	20.00 (10.63–26.50)	
**Grading**			*p* = 0.356
1	14	13.33 (8.375–32.80)	
2	54	20.00 (12.50–28.00)	
3	40	21.00 (12.50–29.50)	
**Histological subtype**			*p* = 0.960
Ductal	90	20.00 (11.81–28.00)	
Other	13	17.50 (12.29–33.50)	
**Molecular type**			*p* = 0.429
Luminal A	30	17.75 (9.69–24.50)	
Luminal B HER2−	25	17.50 (11.13–28.00)	
Luminal B HER2+	22	20.00 (11.81–24.25)	
Non luminal HER2+	5	22.50 (18.00–45.00)	
Triple-negative	14	23.00 (17.00–40.00)	
**ER status**			*p* = 0.311
0	23	22.00 (18.00–40.00)	
1	73	19.00 (11.25–26.50)	
**PR status**			*p* = 0.118
0	26	22.50 (19.50–40.00)	
1	70	17.50 (11.19–28.00)	
**HER2 status**			*p* = 0.563
0	69	20.00 (11.13–28.00)	
1	27	20.00 (14.00–25.00)	

**Table 2:** Hazard ratios (HR) of disease recurrence (DFS) or death (OS) in breast cancer patients from TCGA database focused on the analysis of cytokines overexpressed by ERα36^high^ CAFs. Significant results are bolded.

Cytokine	DFS				OS			
	HR (Hazard Ratio)	95% Lower Cl	95% Upper Cl	Log-Rank *p*-Value	HR (Hazard Ratio)	95% Lower Cl	95% Upper Cl	Log-Rank *p*-Value
CXCL1	1.496	0.155	14.44	0.7	0.8233	0.2375	2.855	0.8
CXCL5	3.98 × 10^−9^	0	Inf	0.3	1.321	0.4677	3.731	0.6
IL-8	4.43	0.6219	31.56	0.1	**3.157**	**1.226**	**8.133**	**0.03**
GM-CSF	3.83 × 10^−9^	0	Inf	0.3	0.4564	0.1046	1.992	0.3
VEGF	1.292	0.1339	12.46	0.8	1.121	0.3651	3.441	0.8
HGF	**7.159**	**0.9431**	**54.34**	**0.03**	**2.697**	**1.025**	**7.094**	**0.04**

## Data Availability

The authors confirm that the data supporting the findings of this study are available within the article [and/or] its Supplementary Materials. Raw expression data were submitted to NCBI GEO database under GSE180186 accession number. Clinical data is not publicly available due to containing information that could compromise the privacy of research participants.

## Supplementary Materials

The following supporting information can be downloaded at https://www.mdpi.com/article/10.3390/cancers14082005/s1, Figure S1: (A) CAF markers analysis by immunofluorescent staining. Vimentin (VIM), E-cadherin (E-CAD), and α-smooth muscle actin (α-SMA); (B) Western blot analysis of CAF markers; Figure S2: 3D Matrigel Hs 578T cell cultures treated with ERα36-negative and ERα36-positive CAF-conditioned media; Figure S3: MDA-MB-231 cell migration through Boyden chamber; Figure S4: 3D Matrigel Hs 578T cell cultures treated with cytokines characteristic of ERα36^high^ CAFs; Figure S5: 3D Matrigel cell cultures of Hs 578T cell line treated with 10 ng/mL HGF or CAF4-conditioned media, with or without 50 nM capmatinib; Figure S6: Western blot analysis of MDA-MB-231 treatment with HGF; Figure S7: Representative pictures of immunohistochemical staining of ERα36 in breast tumor stroma; Figure S8: Overall survival of TNBC patients in our study group (*n* = 15). Files S1: Original images about western blotting. Supplementary Data 1: nCounter PanCancer Immune Profiling Panel results in isolated CAF data. Supplementary Data 2: nCounter PanCancer Immune Profiling Panel results in BC patient data. Supplementary Data 3: Results of GO analysis.
