# Supplementary material for: ERα36-High Cancer-Associated Fibroblasts as an Unfavorable Factor in Triple-Negative Breast Cancer
Source: Cancers (Basel). 2022 Apr 15;14(8):2005. doi: 10.3390/cancers14082005 (PMC9024776; doi:10.3390/cancers14082005)
Supplement: Supplementary file 1 [file cancers-14-02005-s001.zip › cancers-1668878 - suplementary Figure S1-S8, and File S1.pdf]

Supplementary Materials

ERα36-high cancer-associated fibroblasts as an unfavorable factor in triple-negative breast cancer

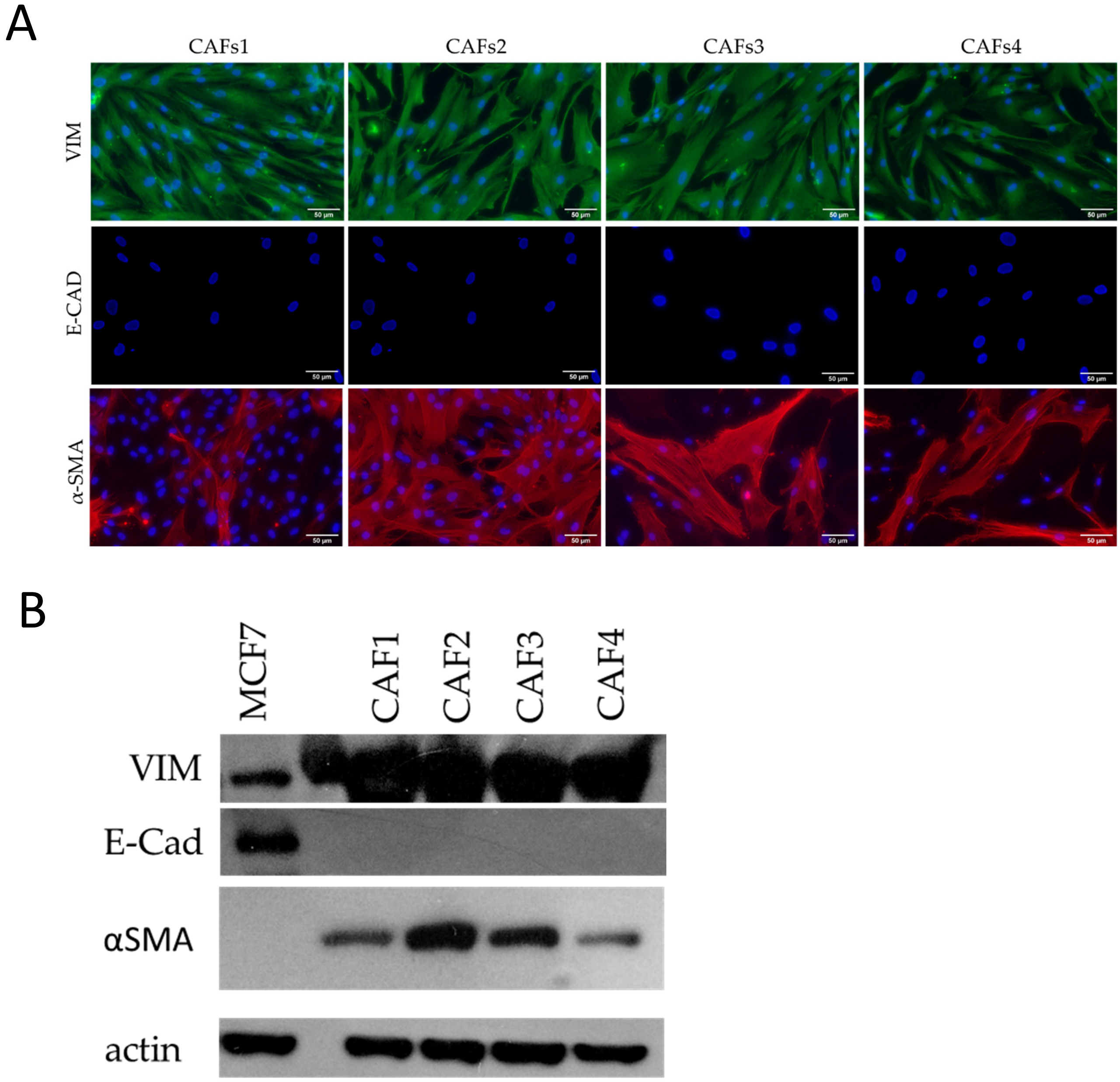

Figure S1. (**A**) CAFs markers analysis by immunofluorescent staining. Vimentin (VIM), E-cadherin (E-CAD) and α-smooth muscle actin (α-SMA); (**B**) Western blot analysis of CAFs markers.

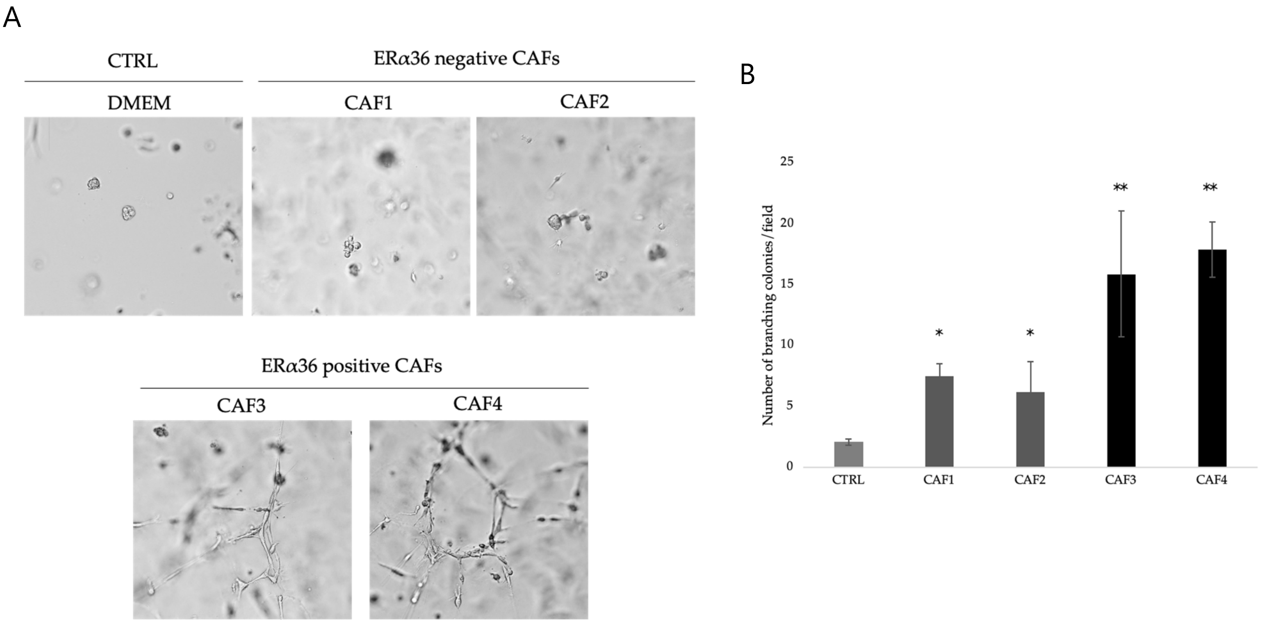

Figure S2. 3D Matrigel Hs 578T cell cultures treated with ER 36^{low} and ER 36^{high} CAFs conditioned media. (**A**) Representative pictures of colonies; (**B**) Graph representation of the results, * $p < 0.001$ ** $p < 0.0001$ in comparison to control. All results were repeated three times independently.

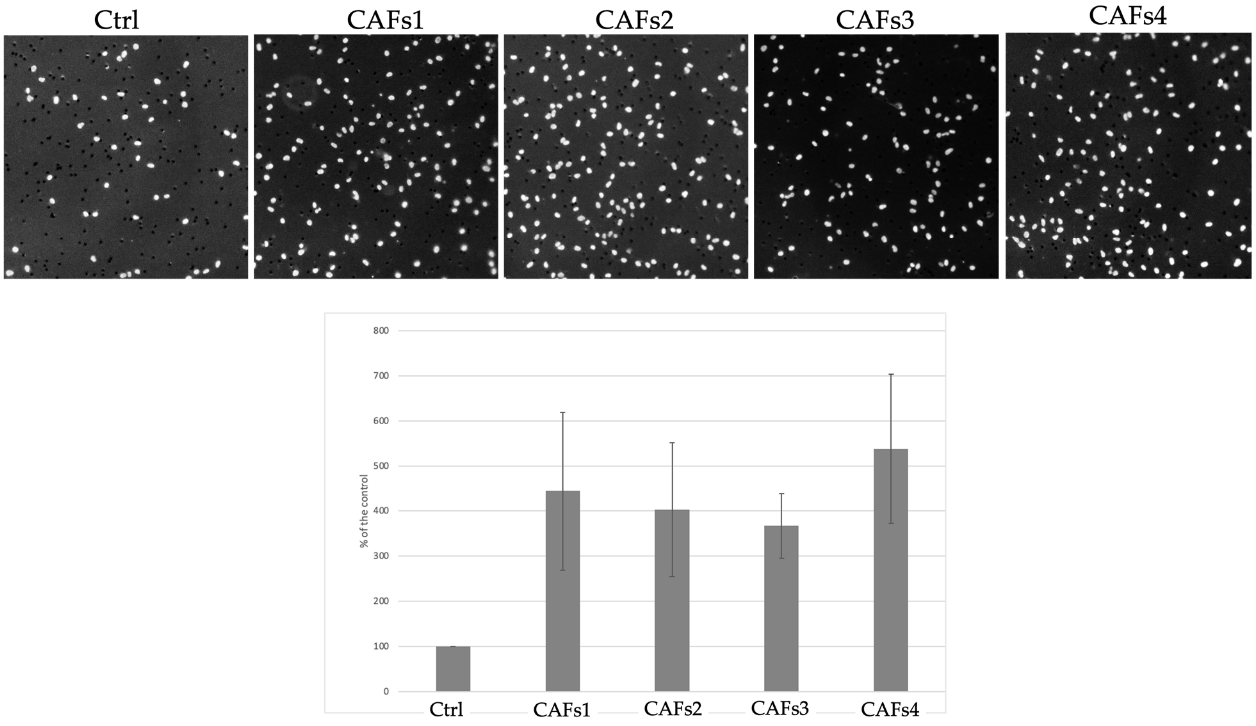

Figure S3. MDA-MB-231 cells migration through Boyden Chamber. No significant differences were observed between CAFs subtypes.

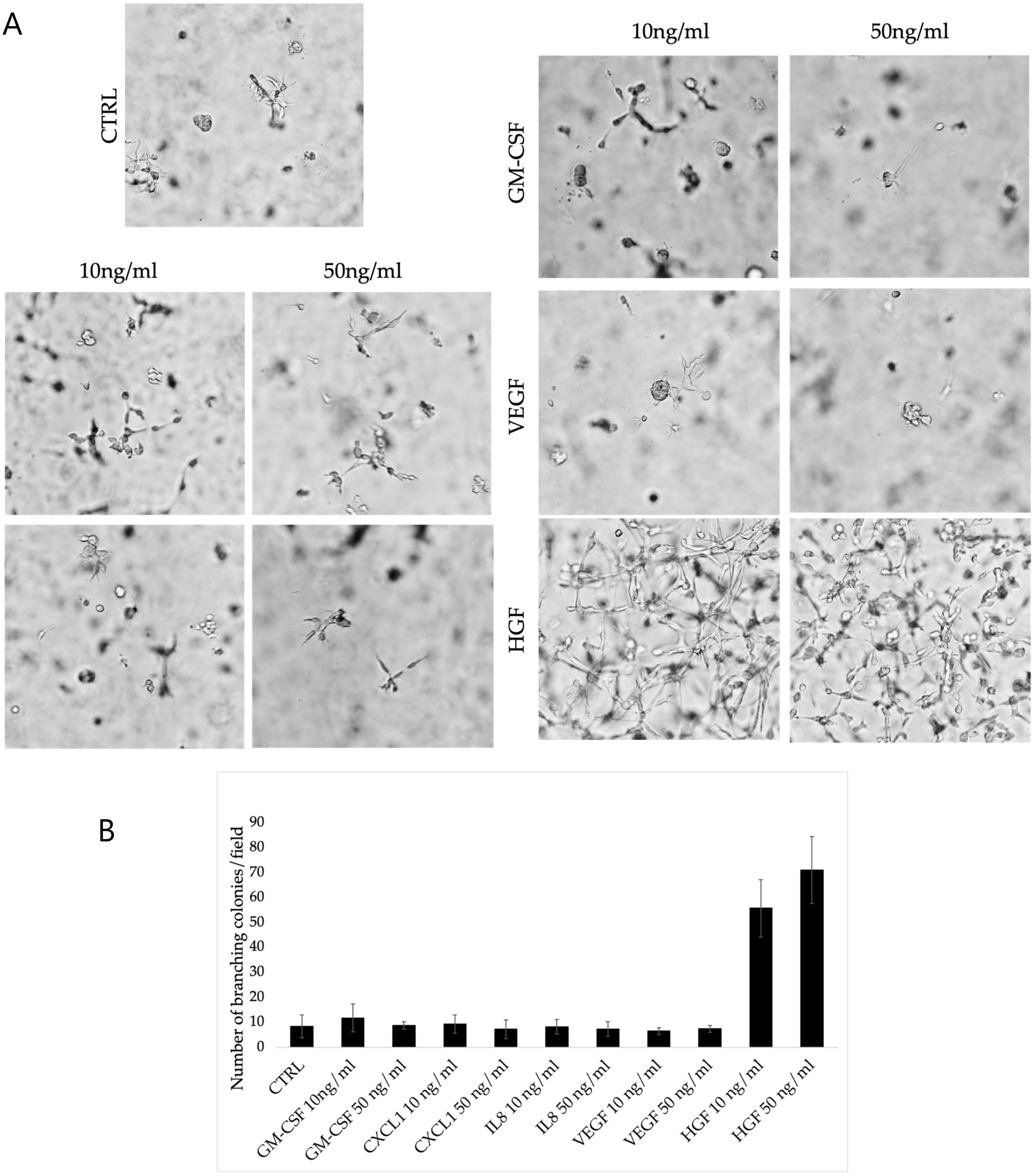

Figure S4. 3D Matrigel Hs 578T cell cultures treated with cytokines characteristic for ER 36[high] CAFs in concentrations of 10 ng/mL and 50 ng/mL. (**A**) Representative pictures of colonies; (**B**) Graph representing of the results, ** $p < 0.0001$ in comparison to control. All results were repeated three times independently.

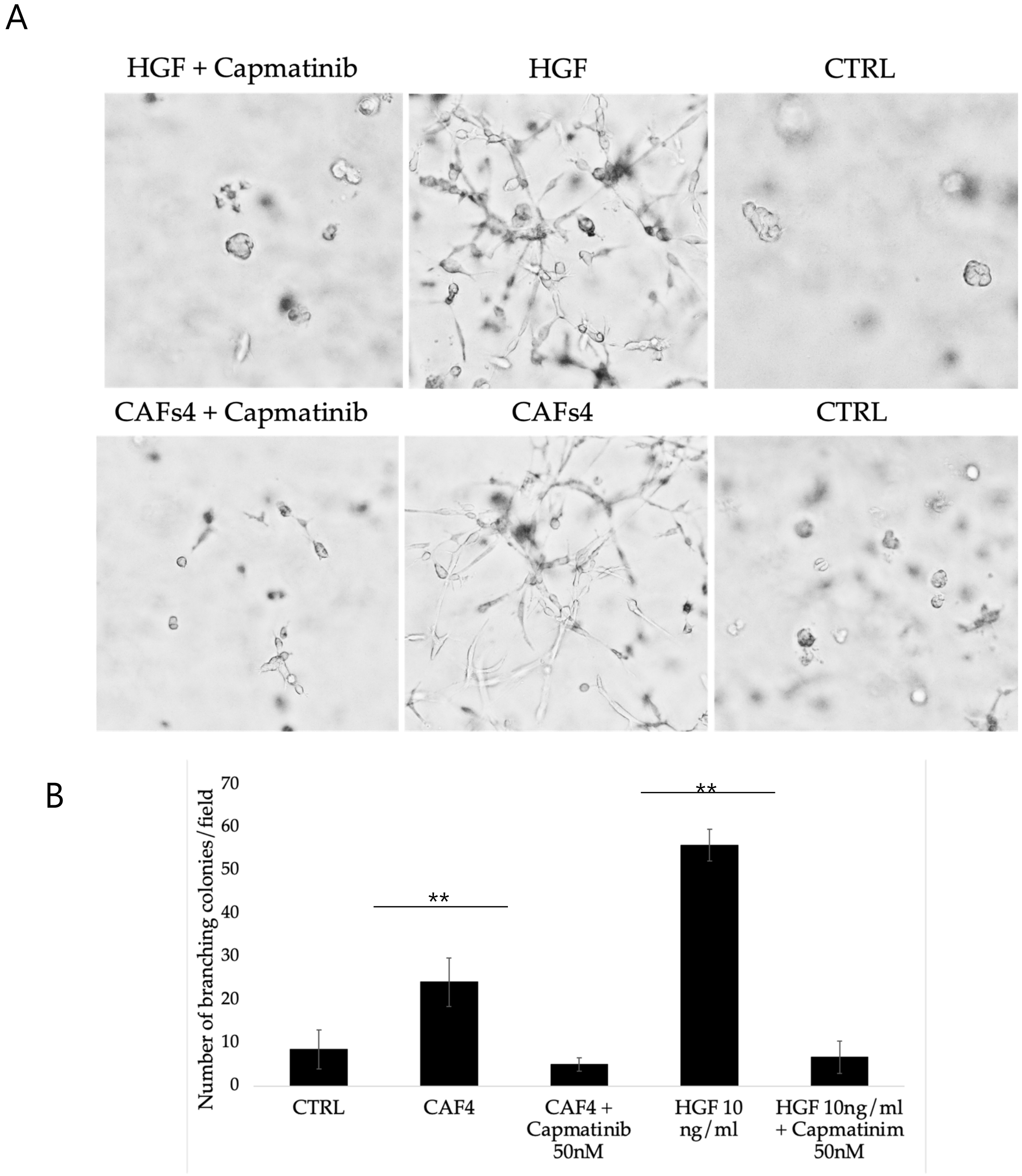

Figure S5. 3D Matrigel cell cultures of Hs 578T cell line treated with 10 ng/mL HGF or CAFs4 conditioned media, with or without 50nM Capmatinib. (**A**) Representative pictures of colonies. (**B**) Graph representing the results, ** $p < 0.0001$. All results were repeated three times independently.

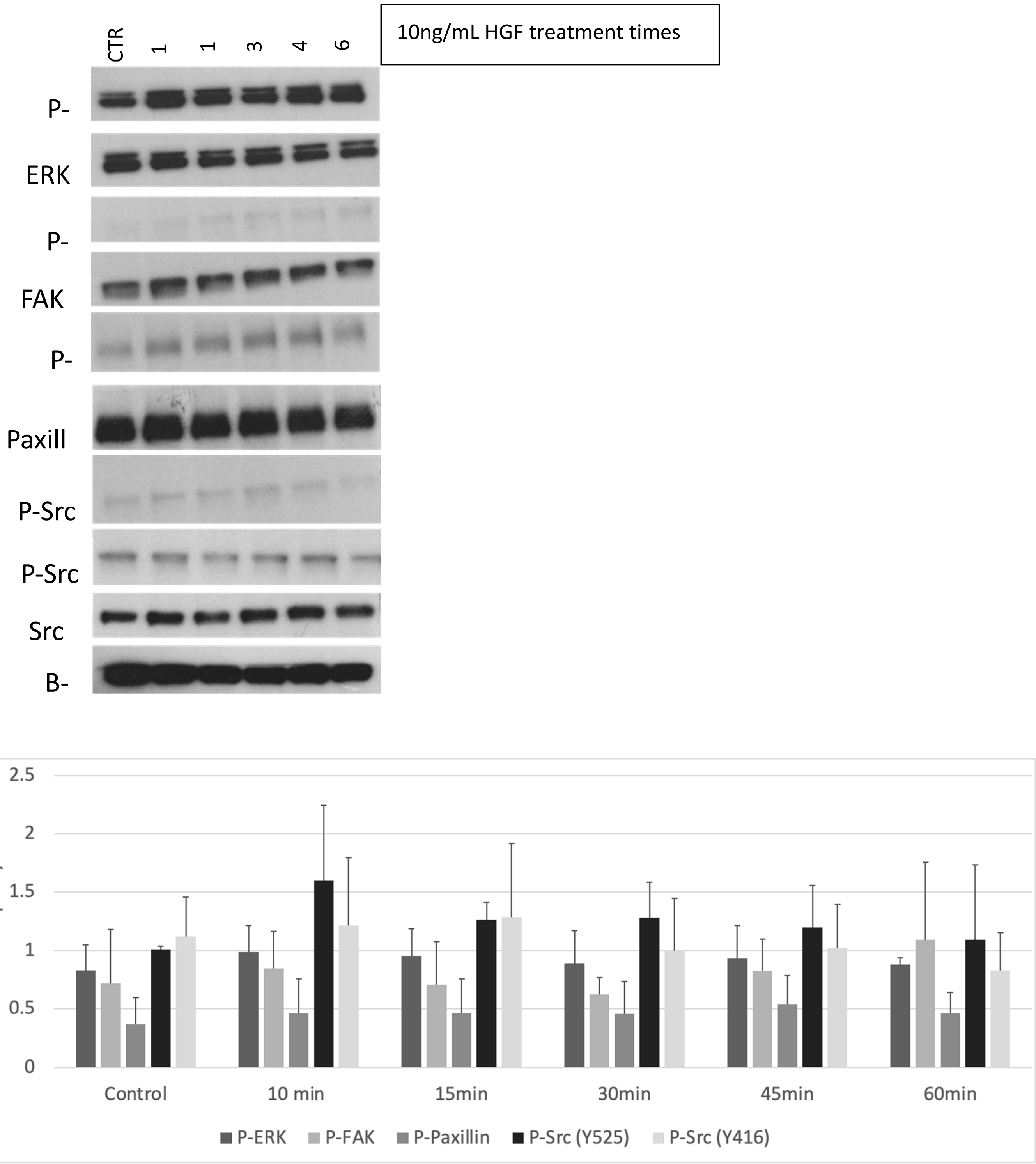

Figure S6. Western blot analysis of MDA-MB-231 treatment with HGF. Bottom graph represents densitometry measurements ratio of phosphorylated to total protein. No differences in the phosphorylation of above proteins was detected.

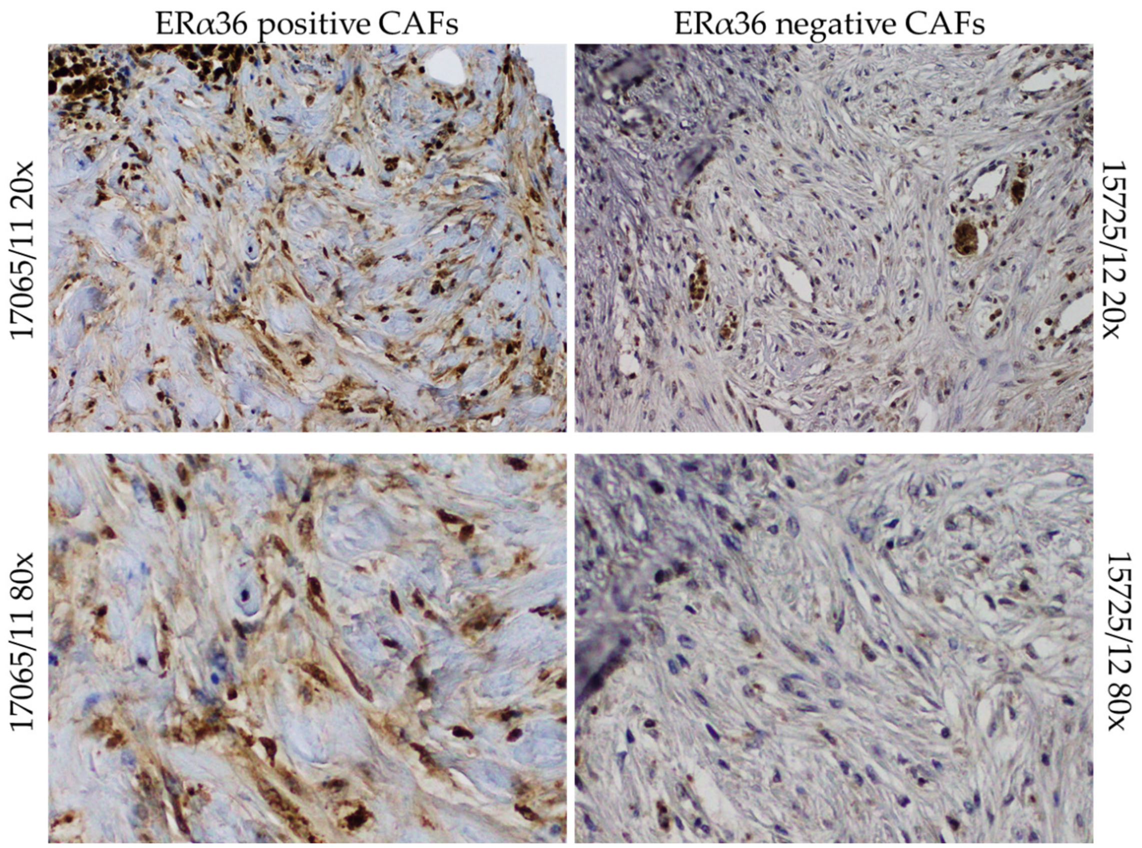

Figure S7. Representative pictures of immunohistochemical staining of ERα36 in breast tumor stroma.

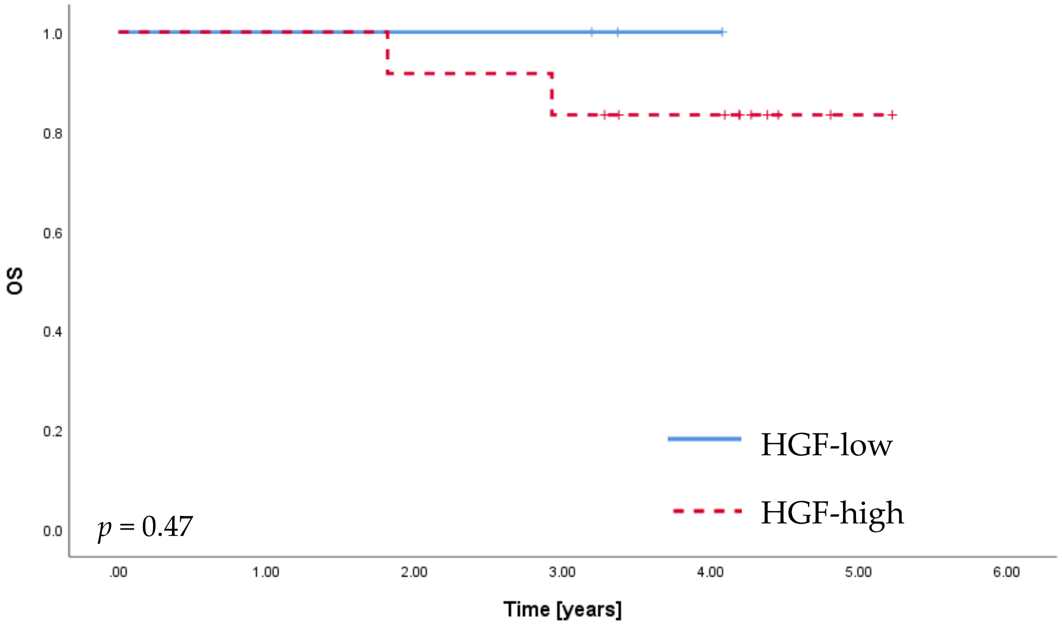

Figure S8. Overall Survival of TNBC patients in our study group (n = 15).

Files S1: Original Images about Western Blotting.

Western blots for Supplementary Figure S1

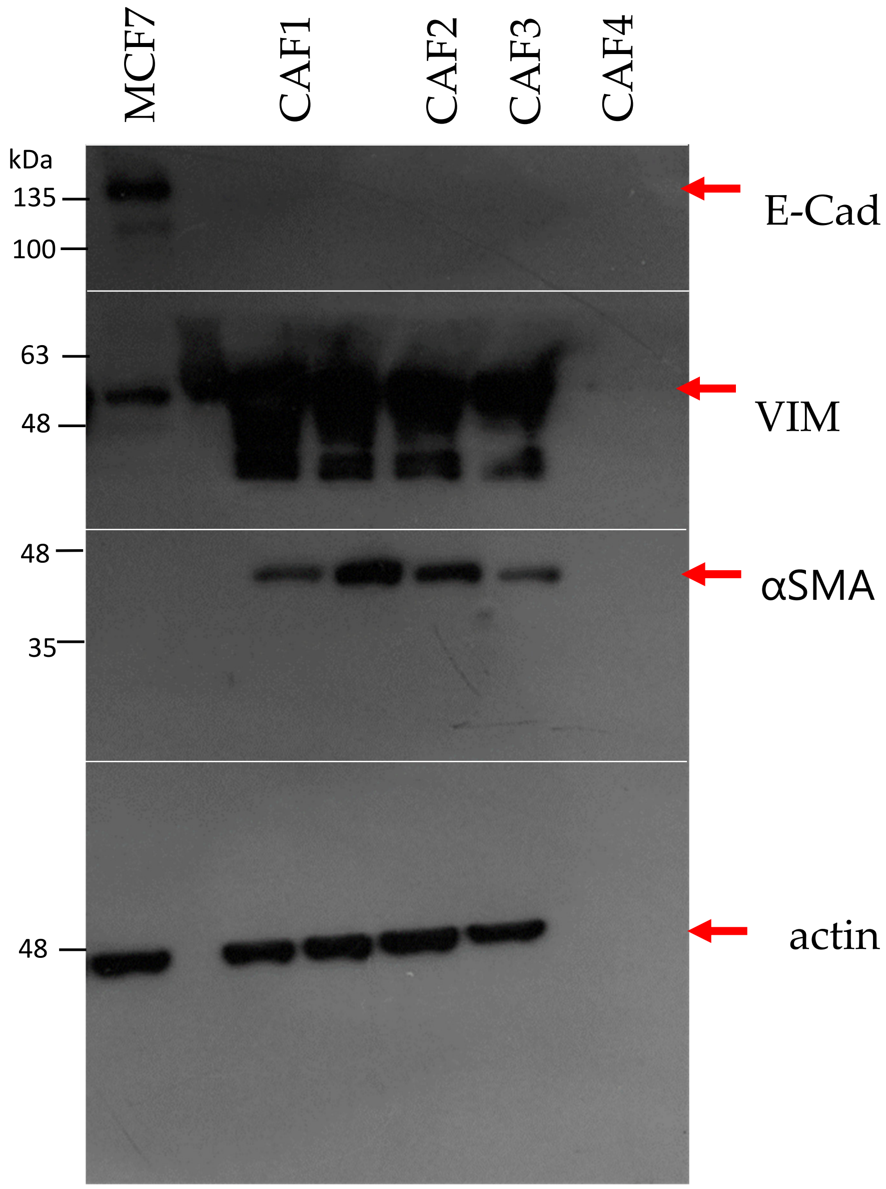

Western blots for MDA MB 231 treatment with HGF (Figure 3). Different films represent different acquisition times, some of the phosphorylated proteins had weak signal and required longer exposure time.

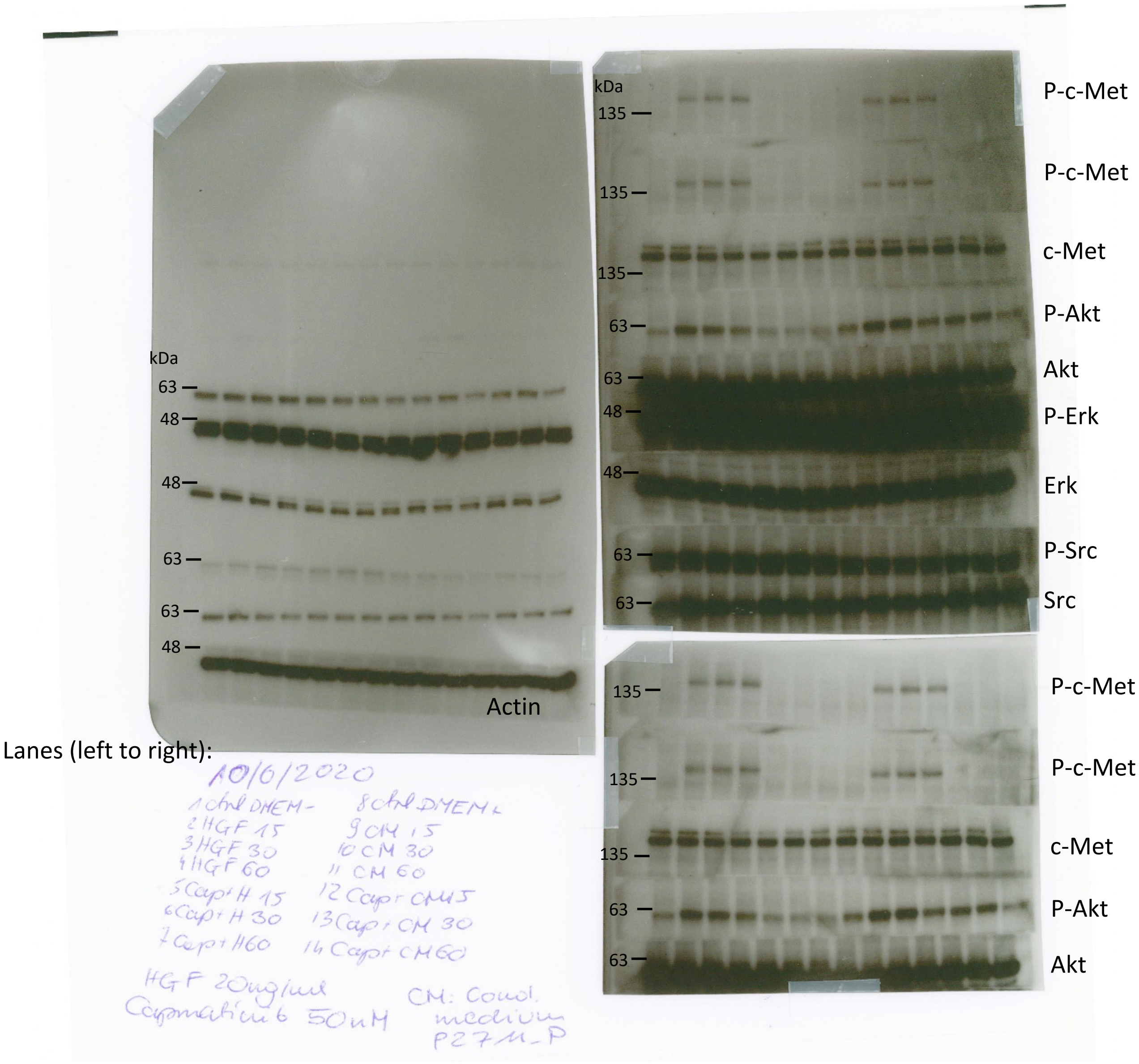

Antibodies:

1 and 2: P-Met
3: Met
4. P-Akt
5.Akt
6. P-ERK
7 ERK
8. P-Src
9. Src
10. Actin

CTRL: MDA MB 231 untreated

CM: Conditioned medium from CAF4 cell line

Cap: Capmatinib

Western blots for MDA MB 231 treatment with HGF and conditioned medium from CAF4 cell line (CM) for Figure 3.

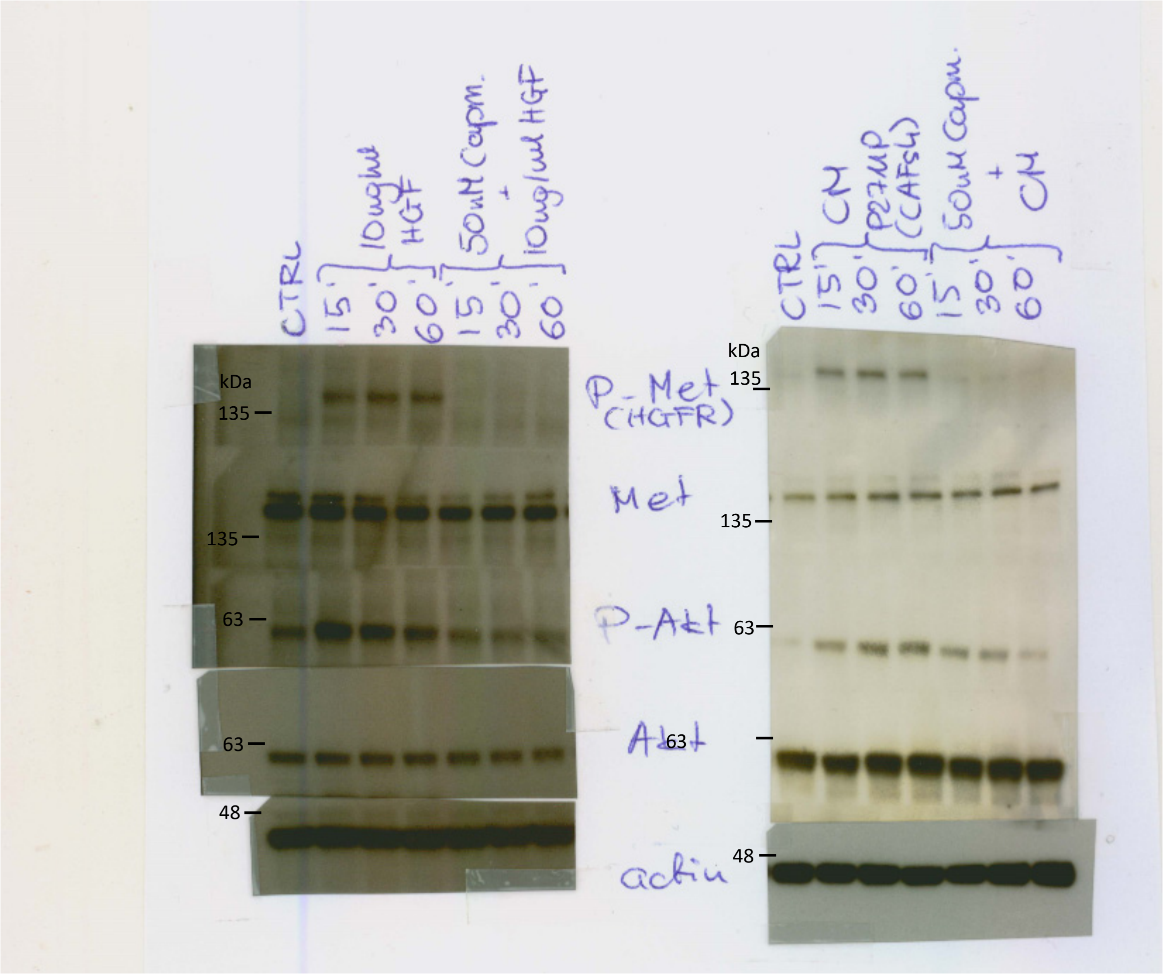

Original Western blot figure for Supplementary Figure S6. Two exposition times.

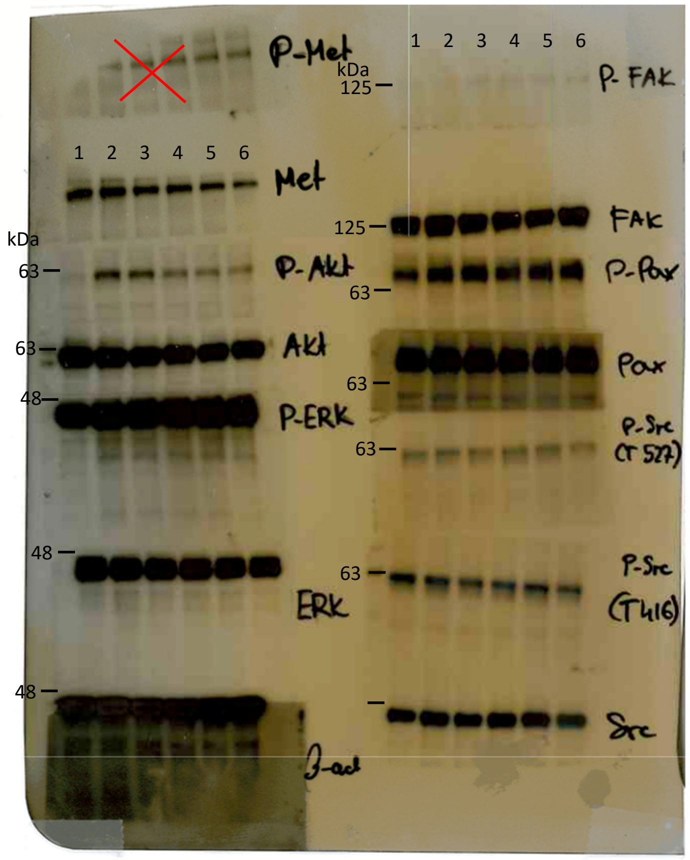

63

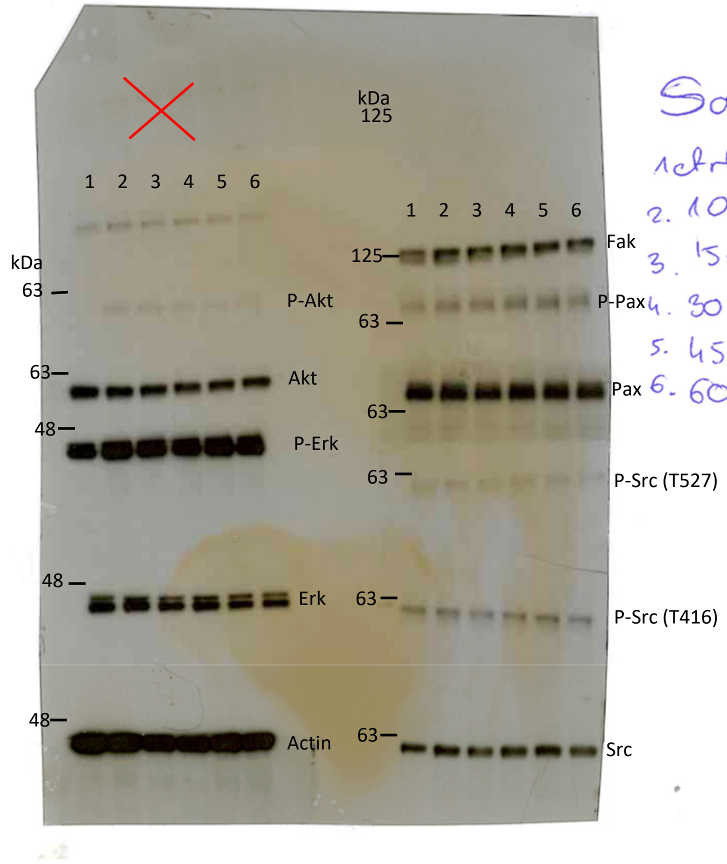

Samples:

1. ctrl
2. 10 min
3. 15 min
4. 30 min
5. 45 min
6. 60 min

HGF 20 ng/ml
Cell starvation 24 h (no FBS)

10/4/2020
